# How do PhD candidates perceive good research practices in the Netherlands Code of Conduct for Research Integrity?

**DOI:** 10.1186/s41073-026-00201-6

**Published:** 2026-06-11

**Authors:** Lodewijk A. Pet, Joeri K. Tijdink, Anna E. van ’t Veer, Ionica Smeets, M. Yvonne H. G. Erkens, Frits R. Rosendaal, Bob Siegerink

**Affiliations:** 1 Department of Clinical Epidemiology, Leiden University Medical Center, Leiden, The Netherlands; 2 Department of Ethics, Law and Humanities, Amsterdam University Medical Center, Amsterdam, The Netherlands; 3 Methodology and Statistics Unit, Institute of Psychology, Leiden University, Leiden, The Netherlands; 4 Science Communication and Society, Leiden University, Leiden, The Netherlands; 5 Department of Labor Law, Leiden University, Leiden, The Netherlands; 6 Directorate of Research Policy, Leiden University Medical Center, Leiden, The Netherlands

**Keywords:** Research integrity, Netherlands code of conduct, Research misconduct, Early-career scientists, PhD candidates

## Abstract

**Introduction:**

Research misconduct and questionable research practices compromise the quality of scientific research. The Netherlands Code of Conduct for Research Integrity 2018 outlines five core principles and 61 standards for good research practices intended to guide researchers and research integrity committees. For the code to function, its 61 standards must be perceived as clear and relevant by those who perform the research practices. These include researchers including PhD candidates, who contribute about 40% of Dutch research full-time equivalents. This study examines how PhD candidates in the Science and Medicine faculties at Leiden University perceive these 61 standards.

**Methods:**

A total of 332 PhD candidates (73% participation proportion) evaluated the standards in the Dutch code as part of a mandatory research integrity course at Leiden University and consented to share their responses for research purposes. Each participant rated half of the standards using Likert-type scales for clarity, relevance, frequency, and seriousness of non-adherence. In addition, open-text responses were collected and categorized according to the code’s five core principles.

**Results:**

Overall, 83% of clarity ratings and 77% of relevance ratings scored 4 or 5, indicating a generally positive perception of the standards. However, the perceived frequency of non-adherence varied across standards (5–34%). There were discrepancies in severity assessments, where standards that should represent minor shortcomings were perceived as serious misconduct and the other way around. Open-text responses primarily emphasized honesty and transparency, while independence was rarely mentioned, and participants also highlighted values not explicitly covered by the current code, such as equality and ethical collaboration.

**Discussion:**

PhD candidates generally regarded the Dutch Code’s 61 standards as clear and relevant, but their ratings of how serious non-adherence should be considered often diverged from the code’s a priori three-tier severity framework. Several standards labelled as “minor shortcomings”, particularly those related to ethics approvals, authorship, and transparent reporting, were frequently classified as research misconduct, while one plagiarism-related standard (practice 34) was more often seen as a questionable practice. These results suggest the need to refine currently ambiguous formulations and reconsider the a priori severity categories.

**Supplementary Information:**

The online version contains supplementary material available at 10.1186/s41073-026-00201-6.

## Introduction

Research misconduct and questionable research practices result in low-quality, unreliable research and research waste [1–3]. In the past decades, there has been a growing emphasis on improving research integrity. This is evidenced by the emergence of research integrity congresses and statements, offices, networks and committees on research integrity, and codes of conduct for research integrity [1, 4, 5]. Despite all this, research practices that distort integrity are still prevalent.

Three meta-analyses, with the most recent one in 2021, report that 2% and 3% of researchers admitted to falsification, fabrication, or plagiarism within the past three years, and 10% to 13% admitted to other questionable research practices. The incidence of observed falsification, fabrication, or plagiarism in colleagues' behavior ranges between 14 and 30% and between 29 and 40% for questionable research practices [6–8]. Dutch researchers are no exception to the occurrence of research misconduct and questionable research practices, as evidenced by the National Survey on Research Integrity conducted in 2020 [9].

In the Netherlands, self-regulation of science is anchored in the Netherlands Code of Conduct for Research Integrity (hereafter, the Dutch code) that is adopted by all Dutch universities and research institutes. This code serves three key purposes: (1) it acts as an educational and normative guide for researchers, trainee researchers, and students, (2) it offers a reference point for research integrity committees when investigating alleged misconduct, and (3) it specifies institutional duties of care [10]. This code builds on five guiding principles of honesty, scrupulousness, transparency, independence, and responsibility, further distilled in 61 standards for good research practices (hereafter, standards).

The Dutch code is part of a global movement to foster research integrity, guided by international frameworks like the ALLEA European Code of Conduct and the Montreal Statement on Research Integrity [11]. While these frameworks set universal ideals, the design and implementation of national codes may vary. Studies have shown differences in the content and structure of codes across Europe, raising questions about clarity and applicability [12]. For other countries, the Dutch code is an informative case: it is based on five core principles, operationalizes these into 61 standards, and specifies how to handle non-adherence to these standards. Many other countries have developed national codes for research integrity with a similar structure. These often align with or draw on broader guidance such as the European code of conduct for research integrity or the Singapore and Montreal statements. The evaluation of how the Dutch framework is perceived by its researchers, offers crucial insights for the international challenge of translating abstract principles into practical, everyday guidance.

The Dutch code was comprehensively updated in 2018 to ensured clearer standards and greater internal coherence, as well as alignment with international documents such as the ALLEA European code of conduct for research integrity, the Singapore statement, and the Danish code of conduct [4, 13, 14]. It is designed as a living document as the current version explicitly anticipated the need for future revisions to ensure its continued relevance. Accordingly, the Royal Netherlands Academy of Arts and Sciences has initiated the next revision to check if the code is still up-to-date and if its standards are practically applicable [15].

For a self-regulation code of conduct, the researchers who are expected to apply the code should be included in the evaluation. Early-career researchers, particularly PhD candidates, are a uniquely valuable population for this evaluation. They represent a large and growing proportion of the scientific workforce in the Netherlands, accounting for 12,445 full-time equivalents (39%) of all university research full-time equivalents in 2024, an increase of 3,953 full-time equivalents since 2018 [16]. Furthermore, their Master’s degree indicates they are equipped with the knowledge to conduct research independently and carry responsibility for their research activities [17]. As the next generation of independent scientists, the inclusion of their perspectives is critical for the code’s standards to be up to date and sustainable.

This study aims to provide empirical evidence for the ongoing revision of the Dutch code of conduct on how the standards of the Dutch code are understood by its users. First, we provide evidence on the practical applicability of the 61 standards by assessing how clear and relevant they are perceived to be by early-career researchers. Second, we inform the discussion on the code's generalizability by empirically testing whether these perceptions differ, even between closely related disciplines such as Medicine and Science. Finally, we contribute to the evaluation of the code’s conceptual foundation by assessing whether its five guiding principles fully encompass the values that PhD candidates themselves identify as most important for good research practices.

To deliver these contributions, our study entails a direct evaluation of several key aspects of the Dutch code. Specifically, we will evaluate:

1. The 61 standards, by quantifying PhD candidates' perceptions across four dimensions: clarity, relevance, estimated frequency of non-adherence, and the perceived seriousness of such non-adherence.
2. The uniformity of these perceptions, by comparing the findings between the faculties of Medicine and Science.
3. The sufficiency of the five guiding principles, by qualitatively analyzing which research practices and values participants formulate themselves and how these align with the current principles.

## Methods

### Study design and participation enrollment

This questionnaire study took place at Leiden University, in the faculties of Science and Medicine. These faculties typically have 450PhD candidates each year, which represents about 75% of all PhD exams at Leiden University [18]. Although distinct, these schools share a largely empirical scientific method, different from other schools, such as humanities or law.

A key challenge in surveys on research integrity is the risk of self-selection bias, particularly when response is low. To mitigate this, we performed the study within an educational activity. The PhD program at Leiden University includes a mandatory research integrity course “Scientific conduct for PhDs” [19, 20]. One part of the preparation was the evaluation of the standards of the code in order to encourage PhD candidates’ critical thinking skills and reflect on what is expected from them as researchers at Leiden University. The questionnaire also served a didactic function, as it helped students prepare for discussion on research integrity during the course. This evaluation environment was changed to an anonymous online questionnaire that could be used for the data collection. Participants were asked to consent to the use of their data for research purposes before they started the questionnaire. Everyone enrolled in this course for the faculties of Science or Medicine was eligible for the study, and all completed questionnaires from individuals who gave consent were included in the final analysis. The course consisted only of participation in the workshop, and there was no exam or individual evaluation.

The timeframe and, with it, the sample size was based on practical circumstances since the Dutch code was up for evaluation near the end of 2023. This approach allowed the committee of the Royal Netherlands Academy of Arts and Sciences to use our results for an advisory about the evaluation of the current Dutch code [21]. The frequency of the planned courses was about two to three per month, with an expected average of 15 to 20 attendees per session. We anticipated a high participation proportion with up to 300 completed questionnaires for data analysis within one year.

A pilot study was performed among several PhD candidates who participated in the workshop just before the start of the study to improve the questionnaire. After three months, the participation rate was reviewed to determine whether the sample strategy required changes, which was not necessary.

### Data collection

Data were collected by changing the existing preparation environment for evaluation the standards into one that gave the option to collect data with an anonymous online questionnaire service provided by Castor Electronic Data Capture (Castoredc, Amsterdam) to ensure the integrity of the data. To mitigate respondent fatigue, we created four questionnaires that covered half of the standards from the code of conduct in a random order. Each standard was randomly assigned so that it appeared twice across the four questionnaire versions, two with 30 items, two with 31 items. To allow for discussion in the workshop, all participants in the same workshop received identical questionnaires. All questions included the option 'Rather not say' or could be left blank. The questionnaire was structured to be completed within 30–45 min and described as a 'one time only' activity. The questionnaires included space to list personal experiences that were not part of the analysis.

Background characteristics were collected on seven different variables (age, place of work, type of work, months of PhD experience, gender, prior research integrity training, and mobility. Mobility was derived from three separate questions; whether 1) the country of origin, 2) the country of getting the bachelor degree and 3) the country of getting the master’s degree was in the Netherlands, in Europe or outside of Europe. These were combined into the categories: 1) Fully educated in the Netherlands, 2) Educated in Europe, 3) partially educated in Europe, 4) educated outside of Europe. Additionally, the time to finish the survey was collected automatically by Castor and was used to evaluate if the preparation for the workshop was still within limits.

To evaluate if a standard can be effective, it should be clear and relevant. Besides directly asking whether a standard is relevant, we also deduced this by measuring perceived frequency and seriousness of non-adherence. A standard might have been perceived as not relevant because its perceived negative impact on research is minor. However, it has been reported that frequently occurring minor misbehaviors can have similar or even greater impact compared with major misbehaviors such as fabrication and falsification of data [22]. Similar reasoning has been applied in prior empirical studies assessing the perceived relevance of research misbehaviors, where both frequency and seriousness were combined as indicators of their overall impact [23, 24].

Participants rated the clarity, relevance, and perceived frequency of non-adherence to individual research practices on a 5-point Likert scale from 1 to 5 (1 representing least clarity relevance and frequency, and 5 the most). For the 5-point Likert scales, only the extreme values 1 and 5 were provided with a label. The values 2 to 4 were placed in between to signify the ordinal scale. The three ordinal labels for non-adherence severity were derived from categories used in the Dutch code. In this study, four research practices directly regarded as research misconduct (fabrication, falsification, and two about plagiarism) were labelled as “research misconduct (most severe)”, a group of 19 practices as “questionable research practice”, and the other 38 practices as “minor shortcomings (least severe)”. These exact labels, including the part between parenthesis, were provided in the questionnaire. However, apart from “least serious” and “most serious”, no further information was provided. Additionally, candidates were instructed not to read the code prior to completion of the questionnaire.

Ordinal categorical measurements were chosen over continuous measurement to reduce response burden. Individual ranking of good research practices per individual was not possible due to the use of 5-point Likert scales and because each participant only evaluated half of the practices.

To assess whether respondents would value practices currently not present in the code, three optional open-text questions were included asking participants to name three, in their view highly important good research practices. These questions were placed immediately after the demographics section, but before the part in which the standards were evaluated. This allowed us to assess whether the five core principles sufficiently capture the values that PhD candidates themselves consider important, while also identifying practices that pointed to potentially missing principles. Based on the description in the Dutch code, the first author (LAP) categorized each response as falling under one or more of the five principles outlined in the Dutch code if possible. Each response was reviewed to see whether it corresponded to one or more of the five Dutch code principles, even if participants did not phrase their answers as formal research practices. Any answer that failed to match one of those five principles, either because it reflected a different principle or was a misinterpretation was grouped under ‘unrelated to the five principles.

### Data analysis and statistics

Continuous data are reported with mean and standard deviation or median with interquartile ranges; categorical variables as proportions with absolute numbers.

To summarize the key findings, four tables list the top 5 most unclear, irrelevant, frequently disregarded, and those of which breaches were considered the most serious infringement according to PhD candidates.

To evaluate the alignment of perceived seriousness of non-adherence with good research practices, the mode was used to label severity of non-adherence to a good research practice as minor shortcoming, questionable research practice, or research misconduct.

To visualize the entire evaluation of the practices, four stacked bar charts delineated all good research practices, and two scatter plots illustrated the relationships between clarity and relevance and frequency and severity. To generate the scatter plots, categorical Likert scale data were interpreted as interval data by assuming equal distances between response categories.

The supplements provide the same data stratified by discipline, an overview of all mean Likert scores per standards, and an interactive version of the data is available at https://zenodo.org/records/15169031, allowing deeper exploration of the findings.

### Ethical approval

The protocol of this study, protocol number A170, was approved by the scientific committee of the Department of Clinical Epidemiology of the Leiden University Medical Center (LUMC). The protocol was preregistered at The Open Science Framework: https://osf.io/db83z. PhD candidates had to consent before and after filling in the questions for their data to be included in the study. A preparatory email for the research integrity course explained the study and that the evaluation of the standards had to be done regardless of participation.

This manuscript differs from the preregistered protocol with these main differences: (1) All responses regarding relevance and frequency were considered, irrespective of clarity scores. (2) We did not assess the consistency among PhD candidates and only focused on the subgroup differences between faculties. (3) Data collection continued until the workshops concluded naturally, rather than stopping at 300 responses.

## Results

### Demographics

**Fig. 1:**
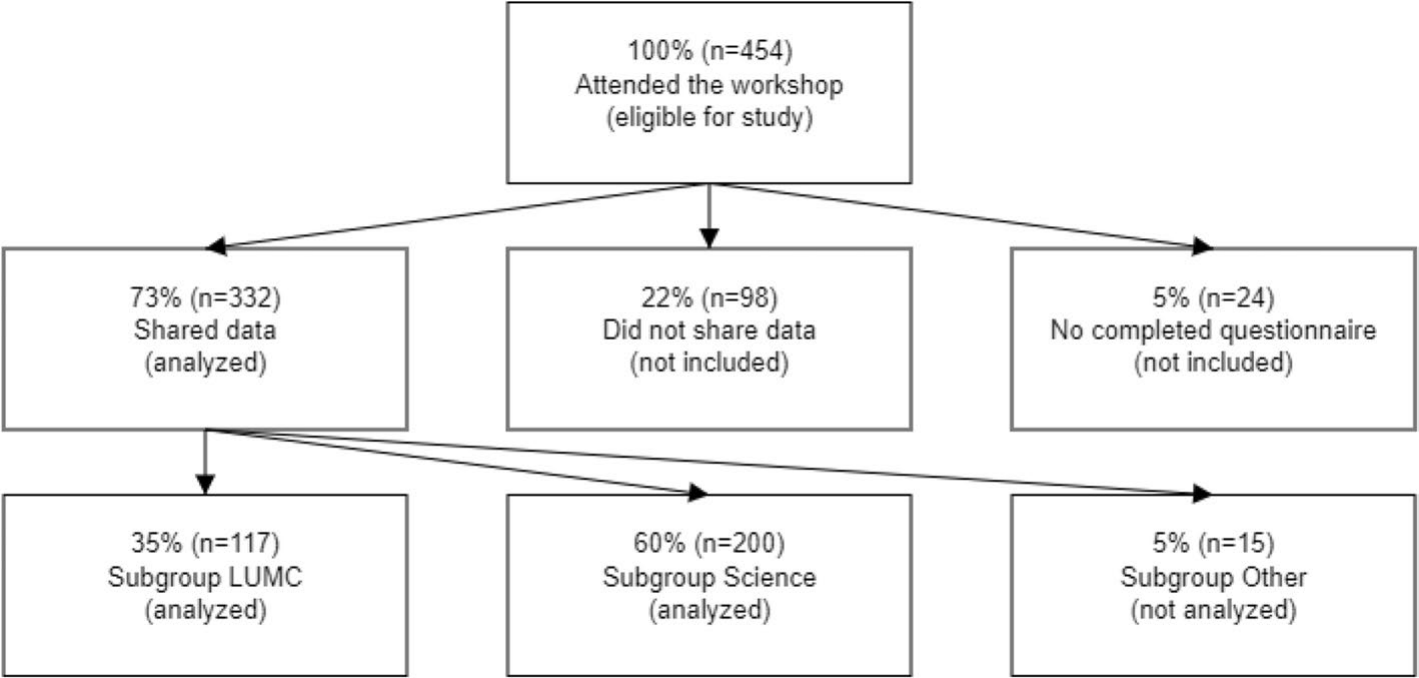
Participation enrollment flowchart. All PhD candidates of the workshop “Scientific conduct for PhDs” from the faculties of Medicine and Science were eligible. In total, 430 questionnaires were completed, whereas 24 participants did not complete the mandatory preparation. There were 95 PhD candidates who followed the link stating they did not agree to share data and then completed the questionnaire. The other 335 candidates followed the link to agree to share their data and completed the questionnaire. However, three of these 335 candidates made use of an additional opt-out option at the end of the questionnaire to not share data. This results in a final 332 participants in this study out of the total 454 eligible participants; and 98 individuals who finished the mandatory preparation for the workshop, i.e. filling in the survey, but did not agree to share data

Between 30 June 2022 and 14 June 2023, a total of 335 PhD candidates completed the questionnaire and consented to share their responses of whom three later decided not to share data, yielding 332 included participants and a participation proportion of 73%. Of these participants, 35% (*n* = 117) were enrolled at the graduate school of Medicine and 60% (*n* = 200) at the graduate school of Science (see Fig. 1).

Among participants from Medicine, there were more women than men (69% women, *n* = 81), compared with 42% women in Science (*n* = 84). A larger proportion of Medicine participants were born and educated in the Netherlands (69%, *n* = 81) than in the Science group (40%, *n* = 80).

Moreover, a greater share of Medicine PhD candidates had fewer than 12 months of PhD experience at the time of the course (62%, *n* = 72), compared with 40% in Science (*n* = 80), i.e., PhD candidates in Medicine attended the course somewhat earlier in their PhD trajectory than their peers in Science.

Missing demographic data amounted to 2%, primarily age with 11% (*n* = 37). Regarding the evaluation of the standards, 3% of all questions were reported as “rather not say”. Among the 332 participants, 6% (*n* = 19) did not attempt to formulate a good research practice in any of the three open text fields. Out of 996 possible answers gathered practices, 88 were left unanswered. Overall, participants from the Science faculty had a slightly higher proportion of missing data than those from Medicine. Table 1 summarizes the background characteristics of the participants.

**Table 1:** Characteristics of all 332 participants

Variable	Faculties of medicine and science	
Age (years)	Measurement	*n* = 295*
	Median [IQR]	27 [25—29]
	Missing	11% (*n* = 37)
Survey time (minutes)	Measurement	*n* = 332*
	Median [IQR]	33 [23—54]
	Missing	0% (*n* = 0)
Mobility**	Group	% (n)
	Fully educated in The Netherlands	52 (172)
	Education in Europe	19 (62)
	Education partially in Europe	9 (30)
	Education outside Europe	16 (54)
Type of work**	Group	% (n)
	Laboratory	34 (114)
	Theoretical	13 (44)
	Clinical	11 (36)
	Computer modelling	10 (33)
	Multiple	24 (80)
Place of work**	Group	% (n)
	Faculty of Medicine	35 (117)
	Faculty of Science	60 (200)
PhD experience**	Group	% (n)
	0–12 months	48 (160)
	13–24 months	28 (94)
	25–36 months	13 (42)
	37–48 months	7 (22)
Gender**	Group	% (n)
	Male	46 (152)
	Female	53 (175)
Prior training**	Group	% (n)
	None	44 (145)
	2 h or less	26 (85)
	Over 2 h	29 (96)

^*^ Total non-missing values

^**^ Groups with less than 5% of observations are not shown

This table presents the demographic characteristics of all 332 participants who evaluated the Good Research Practices in The Netherlands Code of Conduct for Research Integrity (2018. The data are grouped by the faculties of Medicine and Science. Continuous variables are reported as median with interquartile ranges (IQR), while categorical variables are presented as percentages with absolute counts. Missing values are reported as percentages and counts. Mobility combines information on the country of origin, bachelor’s degree, and master’s degree. Survey time refers to the time taken to complete the questionnaire

### Clarity and relevance

The median proportion of participants scoring 4 or 5 was 86% [IQR: 78%−90%] and the median proportion of participants scoring 1 (completely unclear) or 2 was 6% [IQR: 4%−11%] with up to 26% (*n* = 47) of participants scoring the clarity of the practice “*In addressing research misconduct, make no accusation that you know or should have known to be incorrect*” with 1 or 2.

The median proportion of participants scoring 4 or 5 was 82% [IQR: 69%–84%] and the median proportion of participants scoring 1 or 2 was 5% [IQR: 2%–8%]) with up to 34% (*n* = 60) of respondents regarding the practice “*Take on only those tasks that fall within your area of expertise”* irrelevant.

**Fig. 2:**
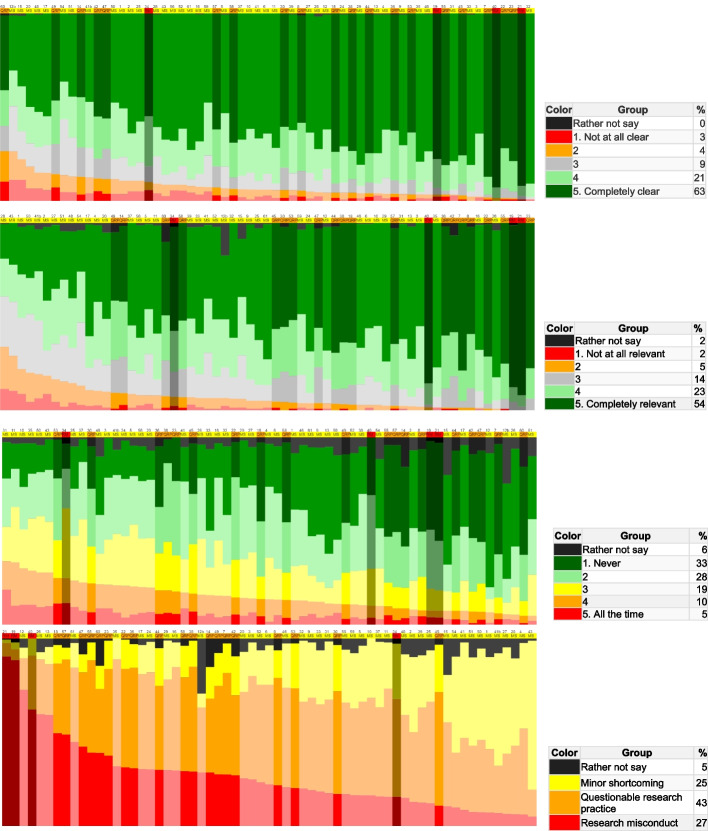
**a**, **b** Ranking the 61 good research practices for good research practices. **c**, **d** The ranking of the 61 good research practices for good research practices based on frequency of non-adherence. Stacked bar charts display evaluations of 61 good research practice standards from “The Netherlands code of conduct on research integrity 2018”. The x-axis shows each practice, with stacked bars indicating the percentage of responses on clarity, relevance, frequency of non-adherence, and seriousness. Bars are ordered by the proportion of high scores (4 or 5—or "Research Misconduct" for seriousness). Practice numbers appear above each bar, with categorization as minor shortcoming (MS), questionable research practice (QRP), or research misconduct (RM) noted below. Bar shading reflects these categories (light for MS, medium for QRP, dark for RM). Standards 12 and 41 are split into “a” and “b,” which result in 63 practices in total

Figure 2a and b visualize the PhD candidates’ ratings in bar charts for clarity and relevance, illustrating that most practices were viewed positively, though a few showed some room for clarification. Table 2 lists the top 5 least clear and relevant practices.

**Table 2:** Top 5 standards with the most extreme scores

**Clarity: To what extent do you understand the research practice that is described in this standard?**				
**Number**	**Standard formulation**	**Chaper 5 category**		**N% answered either "1. Not at all clear” or “2″ on the 5-point Likert scale**
60	In addressing research misconduct, make no accusation that you know or should have known to be incorrect	Questionable research practice		27% (*n* = 47)
12b	In highly exceptional cases, there may be compelling reasons for components of the research, including data, not to be disclosed to an investigation into alleged research misconduct. Such cases must be recorded and the consent of the board of the institution must be obtained prior to using the components and/or data in question in the scientific or scholarly research. They must also be mentioned in any results published	Minor shortcoming		26% (*n* = 50)
15	Enter into joint research with a partner not affiliated with an institution which has adopted this or a comparable Code only if there is sufficient confidence that your own part of the research can be conducted in compliance with this Code and the joint research results meet generally accepted principles of integrity in research	Minor shortcoming		22% (*n* = 47)
20	Do justice to all research results obtained	Minor shortcoming		18% (*n* = 28)
48	Do not use the system of peer review to generate additional citations for no apparent reason, with the aim of increasing your own or other people’s citation scores (‘citation pushing’)	Minor shortcoming		15% (*n* = 23)
**Relevance: To what extent do you think that this research practice should indeed be a standard in The Netherlands code of conduct?**				
**Number**	**Standard formulation**	**Chaper 5 category**		**N% answered either "1. Not at all relevant” or “2″ on the 5-point Likert scale**
28	Take on only those tasks that fall within your area of expertise	Minor shortcoming		34% (*n* = 60)
43	Avoid unnecessary references and do not make the bibliography unnecessarily long	Minor shortcoming		29% (*n* = 48)
1	Consider the interests of science and scholarship and/or society when determining the subject and structure of your research	Minor shortcoming		22% (*n* = 31)
50	Refrain from making an assessment outside your area of expertise, or do so only in general terms	Minor shortcoming		20% (*n* = 33)
41b	Avoid unnecessary reuse of previously published texts of which you were the author or co-author. Such self-citation is not necessary for reuse on a small scale or of introductory passages and descriptions of the method applied	Minor shortcoming		19% (*n* = 34)
**Frequency: How often have you directly seen or experienced non-adherence of this standard?**				
**Number**	**Standard formulation**		**Chaper 5 category**	**N% answered either "5. All the time” or “4″ on the 5-point Likert scale**
31	All authors must have made a genuine intellectual contribution to at least one of the following elements: the design of the research, the acquisition of data, its analysis or the interpretation of findings		Minor shortcoming	34% (*n* = 64)
11	As far as possible, make research findings and research data public subsequent to completion of the research. If this is not possible, establish valid reasons for their non-disclosure		Minor shortcoming	33% (*n* = 47)
10	As necessary, describe how the collected research data are organized and classified so that they can be verified and reused		Minor shortcoming	30% (*n* = 46)
35	Be transparent about the method and working procedure followed and record them where relevant in research protocols, logs, lab journals or reports. The line of reasoning must be clear and the steps in the research process must be verifiable. This usually means that the research must be described in sufficient detail for it to be possible to replicate the data collection and its analysis		Minor shortcoming	29% (*n* = 44)
50	Refrain from making an assessment outside your area of expertise, or do so only in general terms		Minor shortcoming	28% (*n* = 46)
**Seriousness: How would you categorize the seriousness of non-adherence of this standard?**				
**Number**	**Standard formulation**		**Chaper 5 category**	**N% answered "Research misconduct (Most serious violation)"**
21	Do not remove or change results without explicit and proper justification. Do not add fabricated data during the data analysis		Research misconduct	90% (*n* = 150)
19	Do not fabricate data or research results and do not report fabricated material as if it were fact		Research misconduct	88% (*n* = 168)
12a	In the event of an investigation into alleged research misconduct, make all relevant research and data available for verification subject to the confidentiality safeguards established by the board of the institution		Minor shortcoming	73% (*n* = 103)
40	When making use of other people’s ideas, procedures, results and text, do justice to the research involved and cite the source accurately		Research misconduct	62% (*n* = 88)
26	Take into consideration the interests of any humans and animals involved, including test subjects, as well as any risks to the researchers and the environment, while always observing the relevant statutory regulations and codes of conduct		Minor shortcoming	60% (*n* = 106)

The standards are ordered by proportion among three or five options, based on a sample of 332 PhD candidates. The seriousness categories were derived from chapter 5 of the Netherlands Code of Conduct for Research Integrity 2018

**Fig. 3:**
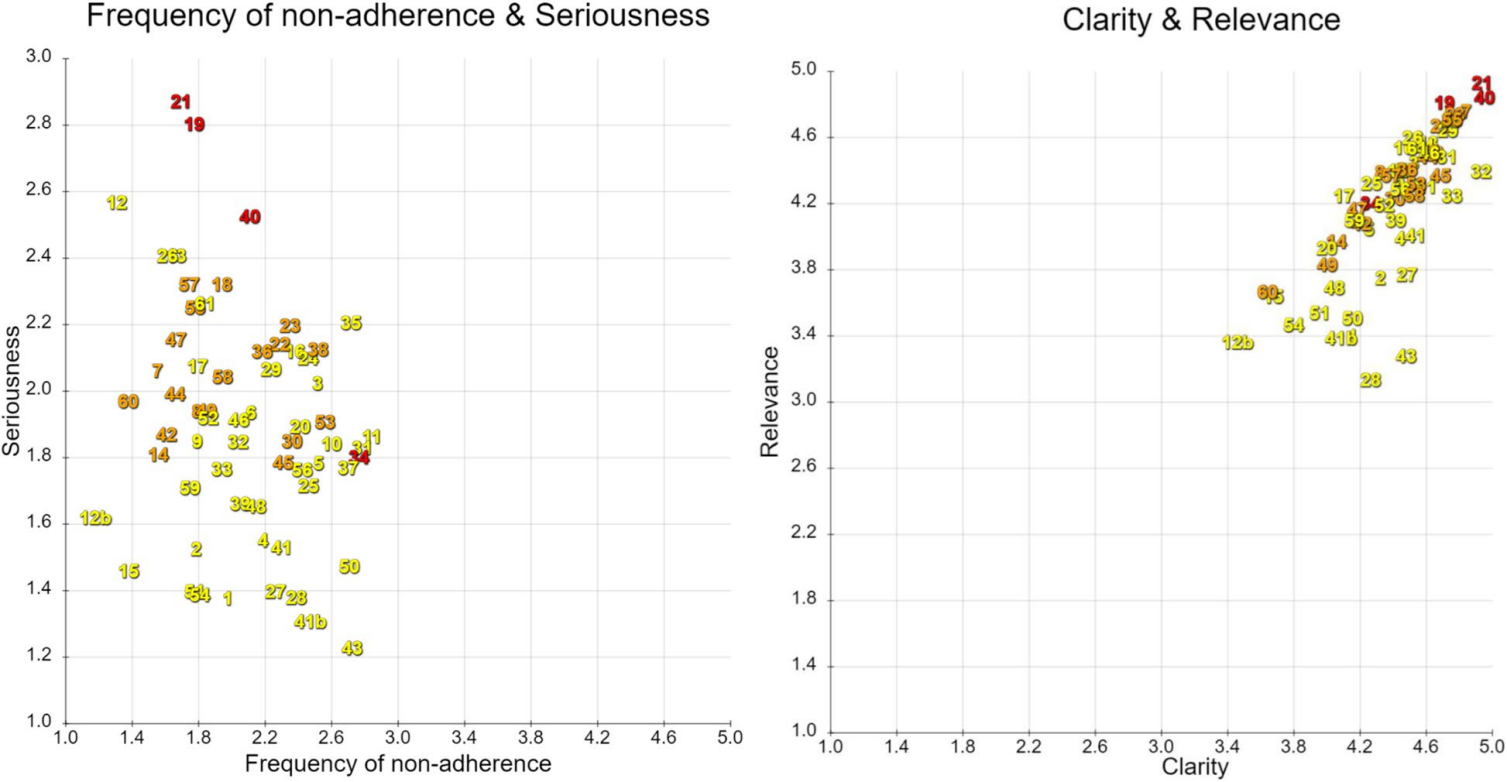
Scatterplots of mean ordinal values of 61 good research practices. The x- and y-axes represent the mean Likert scores for each good research practice in “The Netherlands Code of Conduct for Research Integrity 2018”, calculated after excluding "rather not say" responses. Each practice is represented by a number corresponding to its designation in the Dutch code, with good research practices 12 and 41 subdivided into "a" and "b," resulting in a total of 63 points in the scatter plots. Colors indicate the severity categories from the fifth chapter of the code: yellow for "minor shortcoming," orange for "questionable research practice," and red for "research misconduct." Clarity, relevance, and frequency of non-adherence were measured using a 5-point Likert scale, while seriousness was rated in three ordinal categories: 1 (minor shortcoming), 2 (questionable research practice), and 3 (research misconduct)

The mean 5-point Likert scores of all the standards for clarity was 4.4 (± 0.32) and 4.2 (± 0.43) for relevance ranging between 3.4 and 4.9 for clarity and 3.1 and 4.9 for relevance. The range of the clarity and relevance scores further indicate that the Dutch code consists of standard that are primarily regarded as clear and relevant. The mean Likert scores per practice can be found in supplemental Table s2 and are visualized in the scatterplot in Fig. 3b. This scatterplot shows a positive correlation between mean clarity and mean relevance Likert scores.

### Frequency and seriousness of non-adherence

The median proportion of scores of 4 or 5 was 13% [IQR: 8%–20%] with up to 34% (*n* = 64) for practice 31 that addresses author contribution. Conversely, the median proportion of participants rating the practices as occurring infrequently (scores of 1 or 2) was 63% [IQR: 52%–70%], and for at least one practice, as many as 82% of participants considered it to occur rarely for practice 12b that addresses cooperation in an investigation into alleged research misconduct.

The majority of participants classified three of the four practices explicitly labeled by the Dutch code as definite research misconduct as such. Still, practice 34 “*Present sources, data, and arguments in a scrupulous way* “ was considered a questionable research practice by a majority of 52% (*n* = 74). For the 19 practices that the Dutch code indicates could sometimes constitute research misconduct depending on circumstances, a majority of participants classified five as research misconduct and 14 as questionable research practices. The code states that 38 practices should only be considered research misconduct in exceptional cases, and otherwise as minor shortcomings. The majority of participants identified four as research misconduct, 25 as questionable research practices, and nine as minor shortcomings.

Overall, in majority participants perceived 34 out of the 61 practices (56%) as more serious than indicated by the Dutch code, while only one practice (2%) was viewed as less serious. Figure 2c and d illustrate these discrepancies, highlighting substantial variation between participants' perceptions and the Dutch code classifications. Table 2 lists the top 5 most frequently perceived disregarded practices and those of which infringement was considered as serious.

Figure 2c and d visualize the perceived frequency and seriousness of non-adherence, revealing that perceptions of seriousness of non-adherence varied widely and often differed from the classifications in the Dutch code.

The mean 5-point Likert score for frequency of non-adherence across all standards was 2.1 (± 0.41), ranging from 1.2 to 2.9, all below the middle value of 3. To determine if this is a lot or not depends on the seriousness of the violation. The perceived seriousness of non-adherence by the participants, assessed on an ordinal 1–3 scale, showed considerable variation, with a mean of 1.9 (± 0.35), ranging from 1.2 to 2.9, with few standards that were perceived as very serious. There is no clear indication that PhD candidates estimate that violations they perceive to be more severe occur less frequently than others. Similarly, there is no clear difference in frequency of non-adherence between the practices if we stratify the three categories in the code. The mean Likert scores per practice can be found in supplemental Table s2 and are visualized in the scatterplot in Fig. 3b.

### Falsification, fabrication, plagiarism

The three practices explicitly classified as research misconduct by the Dutch code, 19 “*Do not fabricate data or research results and do not report fabricated material as if it were fact*”, 21 “*Do not remove or change results without explicit and proper justification. Do not add fabricated data during the data analysis*“, and 40 “*When making use of other people’s ideas, procedures, results and text, do justice to the research involved and cite the source accurately*“, were recognized by participants as highly clear, relevant, and serious violations. Specifically, practice 19 (fabrication) was rated as clear by 93% (*n* = 177), relevant by 95% (*n* = 180), and classified as research misconduct by 88% (*n* = 168). Practice 21 (falsification) was rated as clear by 98% (*n* = 162) of participants, relevant by 98% (*n* = 163), and classified as research misconduct by 90% (*n* = 150). Similarly, practice 40 (plagiarism) was considered clear by 99% (*n* = 140), relevant by 97% (*n* = 138), and classified as misconduct by 62% (*n* = 88).

In contrast, research practice 34 *"Present sources, data, and arguments in a scrupulous way"*, which also relates to plagiarism and is classified as misconduct unless the plagiarism is minimal according to the Dutch code, stood out as notably less clear and relevant with 82% (*n* = 116) of participants who considered this practice clear, and 77% (*n* = 110) who considered it relevant. Moreover, only 16% (*n* = 22) classified disregarding practice 34 as research misconduct, while a majority of 52% (*n* = 74) perceived it as a questionable research practice.

Respondents rated practice 34 low on clarity and relevance. They also identified it as misconduct less often than the other three clear practices, which suggests that its description may have downplayed its seriousness.

### Comparison of disciplines

There were a few notable differences related to specific research practices when we compared participants from Medicine and Science. Research practice 40 (plagiarism) was more often considered research misconduct by participants from Science (67%) (*n* = 68) than by those from Medicine (49%) (*n* = 17). Figures s1a to s1h visualize the PhD candidates’ ratings in bar charts for each outcome for the faculties of Medicine and Science separately.

Similarly, Research Practice 31 “*All authors must have made a genuine intellectual contribution to at least of the following elements: the design of the research, the acquisition of data, its analysis or the interpretation of findings*” was reported as the most frequently violated by participants from the faculty of Medicine, where 48% (*n* = 39) rated its non-adherence frequency at 4 or 5 on the Likert scale, compared with 22% (*n* = 22) by participants from the faculty of Science. While medical participants reported non-adherence to this practice more frequently than those from Science, only 11% (*n* = 9) classified non-adherence as research misconduct compared with 24% (*n* = 24) of Science participants.

### Research integrity principles

Participants were asked to list up to three examples of good research practices in their own words, resulting in 908 non-blank responses. Using the definitions for each principle in the Dutch code, for each answer the first author analyzed to which of the five principles it was related: honesty, scrupulousness, transparency, independence, and responsibility. Specifically, honesty was identified in 27% (*n* = 249) of the answers, transparency in 24% (*n* = 219), scrupulousness in 21% (*n* = 195), responsibility in 18% (*n* = 162), and independence in 5% (*n* = 43), with some responses attributed to multiple principles. Additionally, 12% (*n* = 110) of responses could not be related to any of the core principles. They were either misformulations such as research questions, or just did not reflect any of the core principles. Common themes among these uncategorized responses included ‘equality in research’, ‘safe collaboration’ with colleagues, and ‘ethical regulations’.

## Discussion

This study evaluates the perception of the 61 good research practices outlined in the Dutch code by 332 PhD candidates in Medicine and Science on clarity, relevance and the perceived frequency and seriousness of non-adherence. Participants perceive standards as clear and relevant, which shows potential to use the current code for the intended purposes. Every research practice was reported by at least some respondents (≥ 5%) as frequently violated, although these frequency estimates reflect perceptions rather than direct observations. Moreover, ratings of seriousness of breaches of norms displayed a continuous spectrum rather than fitting into discrete categories. These findings suggest that while the practices cover relevant topics, the interpretation of their severity is nuanced and varies considerably.

This study directly sources research practices from the Dutch code, which details 61 practices. Anchoring our evaluation in this operational code allowed us to compare mean Likert scores and prevalence proportions with those reported in previous studies. Mandatory participation of PhD candidates in research integrity workshops led to a high response rate (73%), reducing self-selection bias compared with entirely opt-in surveys. This strengthens the reliability of the results and facilitates robust comparison with prior literature. Our approach offers actionable insights for refining current codes of conduct.

However, these features also limit the comparability of our findings with the existing literature. For instance, while the study among participants of the World Congress on Research Integrity 2015 by Bouter et al. reported average 5-point Likert scores for frequency of observed occurrence of approximately 1.9 for fabrication and 2.2 for falsification, and between 2.7 and 3.1 for plagiarism [23], our study yielded slightly lower scores (1.8 for fabrication,1.7 for falsification, and 2.1 for plagiarism). Furthermore, while the nationwide survey study among Dutch university researchers by Gopalakrishna et al. found prevalence rates of 4.0% for falsification and 5.5% for fabrication [9], our study observed higher rates of 7.2% and 7.5%, respectively. These discrepancies likely stem from methodological variations, such as differences in sampling design (e.g., self-selected conference participants versus mandatory course participants), the populations studied (senior versus early-career researchers), and the framing of questions (general misbehavior versus evaluation of specific code standards).

### Mismatch in severity classifications

Respondents frequently disagreed with the code’s classification of certain practices that only in exceptional cases of non-compliance are to be characterized as research misconduct. For example, non-adherence to research practices 12, 13, 26, and 61, addressing, respectively, participation in misconduct investigations, obtaining ethical approvals, balancing all stakeholders’ interests, and avoiding improper research use, was each perceived as research misconduct by over 45% of participants, contrasting the code’s categorization where only in exceptional cases non-compliance is to be characterized, in the light of the assessment criteria, as research misconduct.

Most respondents classified fabrication (practice 19) and falsification (practice 21) as research misconduct, a minority (12% and 10%, respectively) did not. We interpret this not as condoning such behaviors, but rather as an indication that some respondents may have misunderstood the descriptions, responded inattentively, or were influenced by acquiescence bias. Nevertheless, the fact that not all participants unequivocally recognized fabrication and falsification as the most severe form of misbehavior, underscores the importance of clear and precise wording of these standards in codes of conduct.

Practices 34 and 40 both address plagiarism; however, PhD candidates regarded non-adherence to 34 as less severe than to 40. This discrepancy strongly suggests that the current wording of practice 34 leads to confusion about its intended severity, ultimately resulting in its underestimation by early-career researchers. Therefore, a more explicit delineation of the specific behavior that constitutes plagiarism may be warranted.

More broadly, the code’s a priori three-tier classification does not reflect the continuum of perceived severity, even when respondents were constrained to use three categories. The code mixes two types of standards: those that are classified as research misconduct and those that should be considered misconduct only in exceptional cases. We therefore recommend re-evaluating how the standards are classified or, alternatively, reconsidering how the classes are labelled or derived.

### Differences between disciplines

Although Medicine and Science PhD candidates converged in most evaluations, a few research practices revealed notable differences. For instance, a higher proportion of medical participants deemed non‐adherence to practice 31 (on proper authorship allocation) more frequent than Science participants, while Science participants were more likely to classify non‐adherence to practice 40 (on citing others’ ideas) as research misconduct. One possible explanation is that these groups have distinct disciplinary practices around authorship and citation, yet the modest sample per practice also raises the chance of false positive results. Furthermore, the possibility of further differences with other disciplines such as humanities or social sciences cannot be excluded based on these findings.

### Limitations

The Dutch code stipulates in Sect. 5.2C that final classification of a breach always requires a case-specific assessment and deliberation. Our study does not and cannot evaluate such contextual judgments. Instead, it investigates perceptions of the code’s a priori, context-free grouping of standards.

Our sample was limited to two disciplines (Medicine and Science), restricting generalizability. Although the small differences between these disciplines suggest consistency, more significant variation might emerge across disciplines such as humanities or law.

Due to sequential evaluations, cognitive biases, such as anchoring, learning, and fatigue effects, could have influenced ratings. Randomization of item order and splitting standards across questionnaire versions helped mitigate these biases.

Our analysis relies on treating ordinal Likert responses as approximately continuous, an assumption sensitive to skew and floor/ceiling effects [25]. While anchoring descriptors helped reduce such effects for most items, the three seriousness-of-breach categories could not be similarly anchored and their uniformly negative wording likely intensified floor/ceiling patterns. These ratings should not be interpreted as absolute severity measures.

### Implications and future directions

Preliminary findings were presented to an advisory committee of the Royal Netherlands Academy of Arts and Sciences, whose evaluation of the code benefited from this evidence [26]. The observed inconsistencies, especially around plagiarism definitions and practices requiring ethical approvals, underscore the urgency of clarifying the code’s wording and a priori severity categories.

Future research might focus on the influence of context regarding the change of perceptions. Are the a priori categories really that strict that non-adherence to certain standards indeed only in rare cases will be regarded as research misconduct?

Additionally, when participants formulated good research practices in their own words, their responses primarily reflected the principles of honesty, transparency, and scrupulousness outlined by the Dutch code, while independence appeared less prominently. Since the committee already declared to remain with the same core principles [21], some section in the code might highlight the relationship between the research integrity principles in relationship with the identified themes, equality, safe collaboration, and broader ethical considerations.

Survey studies with similar methods could be conducted in other schools and institutions within and beyond the Netherlands. They might incorporate continuous scales or alternative ranking methods for more nuanced insight into how researchers prioritize different practices. Stratified analyses (e.g., by academic rank or international background) could further refine our understanding of which standards need more explicit definitions or discipline‐specific examples. Additionally, qualitative follow‐up (e.g., interviews or focus groups) might enrich our grasp of why certain good research practices seem confusing or irrelevant and how best to address these gaps.

Like the Dutch code, many national research integrity codes are based on a similar set of core principles that are translated into operational standards for good research practice. We identified several standards that were interpreted differently by users than intended by the code’s designers. Similar mismatches may also be present in other national or institutional contexts. This is particularly relevant for widely accepted forms of research misconduct such as plagiarism, for which we observed discrepancies in perceived seriousness. Prior work also suggests that the perceived detrimental impact of plagiarism-related misbehaviors can differ across disciplinary fields [27]. When developing or revising a code of conduct, it is therefore important to incorporate the perceptions of those who apply its standards in practice.

## Conclusion

Our study indicates that PhD candidates generally find the 61 good research practices in the Netherlands Code of Conduct for Research Integrity 2018 clear and relevant. However, there is a significant misalignment between their perceptions of non-adherence severity and the code's existing severity framework. Several practices officially labeled as minor shortcomings, notably those related to ethical approvals, stakeholder interests, and transparent reporting, were considered serious misconduct. Additionally, ambiguities around plagiarism (practice 34) highlight the need for more straightforward, explicit language. To ensure the standards in the Dutch code can be effective, we recommend to revise the a priori severity classification and clarifying the wording of standards identified as confusing or misinterpreted.

## Supplementary Information

Supplementary Material 1.

Supplementary Material 2.

## Data Availability

All data regarding evaluations of the good research practices can be found online at https://zenodo.org/records/15169031. Baseline characteristics data and open-text question are available on reasonable request.
