# Supplementary material for: How do PhD candidates perceive good research practices in the Netherlands Code of Conduct for Research Integrity?
Source: Res Integr Peer Rev. 2026 Jun 11;11:17. doi: 10.1186/s41073-026-00201-6 (PMC13255387; doi:10.1186/s41073-026-00201-6)
Supplement: Supplementary file 1 — Supplementary Material 1. [file 41073_2026_201_MOESM1_ESM.docx]

# STROBE Checklist — Cross-Sectional

| **Item** | **Recommendation** | **Explanation** | **Page** |
| --- | --- | --- | --- |
| 1a | Indicate the study design with a commonly used term in the title or abstract. | We identify the study as a cross-sectional survey in the Abstract and describe the questionnaire design up front. | 1 |
| 1b | Provide an informative and balanced summary in the abstract. | We summarise the context, aims, methods (setting, participants, measures), and the main findings with a concise interpretation in the Abstract. | 1 |
| 2 | Explain the scientific background and rationale. | We outline the rationale for examining perceptions of good research practices within the Dutch Code, situating it in the broader integrity landscape in the Introduction. | 3-4 |
| 3 | State specific objectives and any prespecified hypotheses. | We state clear objectives focused on describing PhD candidates’ perceptions and mapping them to guiding integrity principles; no formal hypotheses were prespecified for inferential testing. | 4 |
| 4 | Present key elements of study design early in the paper. | We describe the cross-sectional, questionnaire-based design at the start of Methods, including who was invited and how data were captured. | 4-6 |
| 5 | Describe the setting, locations, and relevant dates. | We specify Leiden University (Faculties of Science and Medicine) as the setting and report the data collection window and course context in Methods. | 4 |
| 6a | Give eligibility criteria and sources/methods of selection of participants. | We detail eligibility as enrolment in a mandatory research integrity course and explain the invitation and consent procedure in Methods. | 4 |
| 7 | Clearly define all outcomes, exposures, predictors, potential confounders, and effect modifiers. | We define our primary descriptive outcomes (clarity, relevance, frequency, and perceived seriousness of non‑adherence; open-text principles) and list background variables captured for subgroup descriptions. | 5-6 |
| 8* | For each variable, give data sources and assessment methods; describe comparability if >1 group. | We describe the questionnaire items, scales (e.g., Likert), and administration method; both faculties received the same instrument and procedures. | 5-6 |
| 9 | Describe efforts to address potential sources of bias. | We mitigate self‑selection by embedding the survey in a mandatory course, randomise item order, and include ‘rather not say’ options; limitations are discussed explicitly. | 4-6 |
| 10 | Explain how the study size was arrived at. | We used a census approach: the study size was determined by the number of eligible course participants in the data collection period; no formal power calculation was required for our descriptive aims. | 4-5 |
| 11 | Explain handling of quantitative variables. | We report how Likert responses and three‑level seriousness were summarised and how any categorical groupings were defined a priori. | 5-6 |
| 12a | Describe all statistical methods, including those used to control for confounding. | We specify descriptive statistics and any simple comparative summaries; confounder adjustment was not applicable given the descriptive objectives. | 6 |
| 12b | Describe any methods used to examine subgroups and interactions. | We predefine subgroup descriptions by faculty/discipline and present comparative summaries where informative. | 6 |
| 12c | Explain how missing data were addressed. | We describe handling of missing/‘rather not say’ responses and report missingness per variable; no imputation was performed. | 5 |
| 12d | If applicable, describe analytical methods accounting for sampling strategy. | Not applicable; we did not employ a complex sampling design. | na |
| 12e | Describe any sensitivity analyses. | None were required for the descriptive objectives. | na |
| 13a* | Report numbers of individuals at each stage of the study. | We report the number invited/eligible, the number who consented/started, and the number included in analyses. | 8 |
| 13b* | Give reasons for non-participation at each stage. | We state reasons such as non‑consent or incomplete responses where known. | 8 |
| 13c* | Consider use of a flow diagram. | We include a simple participation flow to visualise inclusion and analysis numbers. | 20 |
| 14a* | Give characteristics of study participants and information on exposures/confounders. | We present participant demographics and background variables in a baseline table. | 17 |
| 14b* | Indicate number of participants with missing data for each variable. | We report missing counts/percentages alongside each baseline variable and key outcomes. | 17 |
| 15* | Report numbers of outcome events or summary measures. | We provide summary measures (proportions/means) for the main outcomes and list top‑ranked items with accompanying counts. | 8-10 |
| 16a | Give unadjusted (and, if applicable, adjusted) estimates with precision; state confounders. | We provide unadjusted descriptive estimates with denominators; no adjusted analyses were prespecified. | 8-10 |
| 16b | Report category boundaries when continuous variables were categorized. | We specify scale ranges (e.g., 1–5 Likert) and the thresholds used for any categorizations (e.g., ‘agree’ defined as ≥4/5). | 8-10 |
| 16c | If relevant, translate estimates of relative risk into absolute risk. | Not applicable; no risk estimates are presented in a cross‑sectional descriptive survey. | na |
| 17 | Report other analyses done (e.g., subgroups, interactions, sensitivity). | We indicate any additional comparative summaries or robustness checks and provide references to supplementary materials if applicable. | 10 |
| 18 | Summarise key results with reference to study objectives. | We open the Discussion by restating the objectives and linking the principal findings to each objective. | 11 |
| 19 | Discuss limitations of the study, considering potential bias/imprecision. | We discuss selection and information biases, scale limitations, and contextual dependence, including likely direction and magnitude of their impact. | 12 |
| 20 | Provide a cautious overall interpretation of results considering objectives, limitations, multiplicity, and relevant evidence. | We interpret findings conservatively, relate them to prior literature and the Code, and avoid overgeneralisation beyond the study frame. | 13 |
| 21 | Discuss generalisability (external validity). | We describe generalisability limits (two faculties at one university) and where findings may or may not transfer. | 21, 22 |
| 22 | Give the source of funding and the role of the funders. | We state the funding source and confirm the funder had no role in design, analysis, or reporting. | 14 |
