# Supplementary material for: How do PhD candidates perceive good research practices in the Netherlands Code of Conduct for Research Integrity?
Source: Res Integr Peer Rev. 2026 Jun 11;11:17. doi: 10.1186/s41073-026-00201-6 (PMC13255387; doi:10.1186/s41073-026-00201-6)
Supplement: Supplementary file 2 — Supplementary Material 2. [file 41073_2026_201_MOESM2_ESM.docx]

**
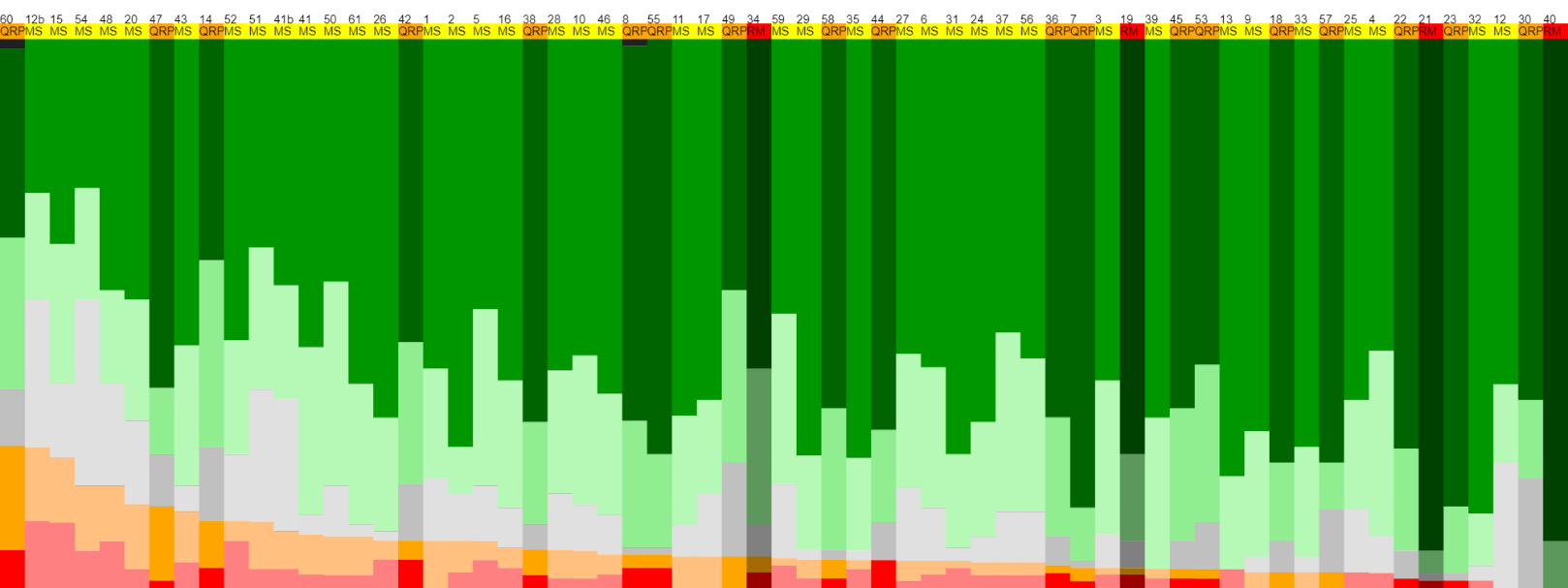
Figure s1 a&b. Likert scores on clarity of 61 Good Research Practices by the faculty of Medicine (a) and Science (b)**

| **Color** | **Group** | **%** |
| --- | --- | --- |
|  | Rather not say | 0 |
|  | 1. Not at all clear | 3 |
|  | 2 | 4 |
|  | 3 | 9 |
|  | 4 | 21 |
|  | 5. Completely clear | 63 |

| **Color** | **Group** | **%** |
| --- | --- | --- |
|  | Rather not say | 0 |
|  | 1. Not at all clear | 3 |
|  | 2 | 4 |
|  | 3 | 9 |
|  | 4 | 18 |
|  | 5. Completely clear | 65 |

**
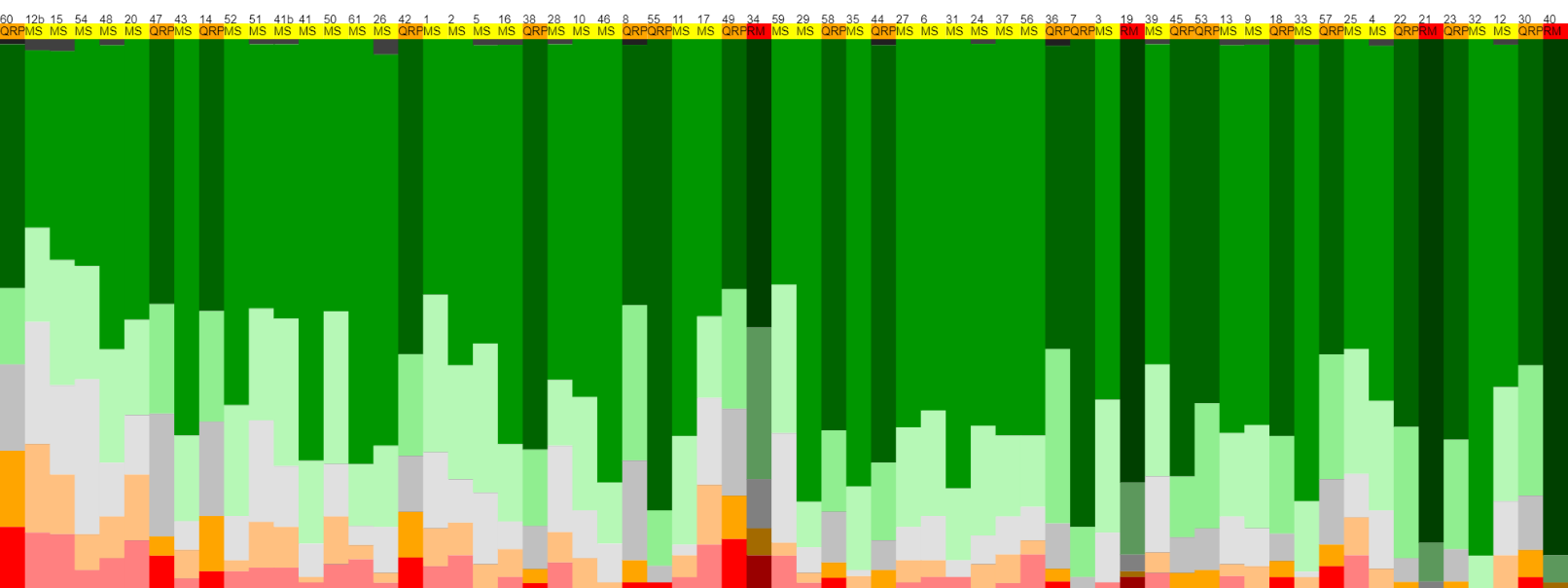
**

**
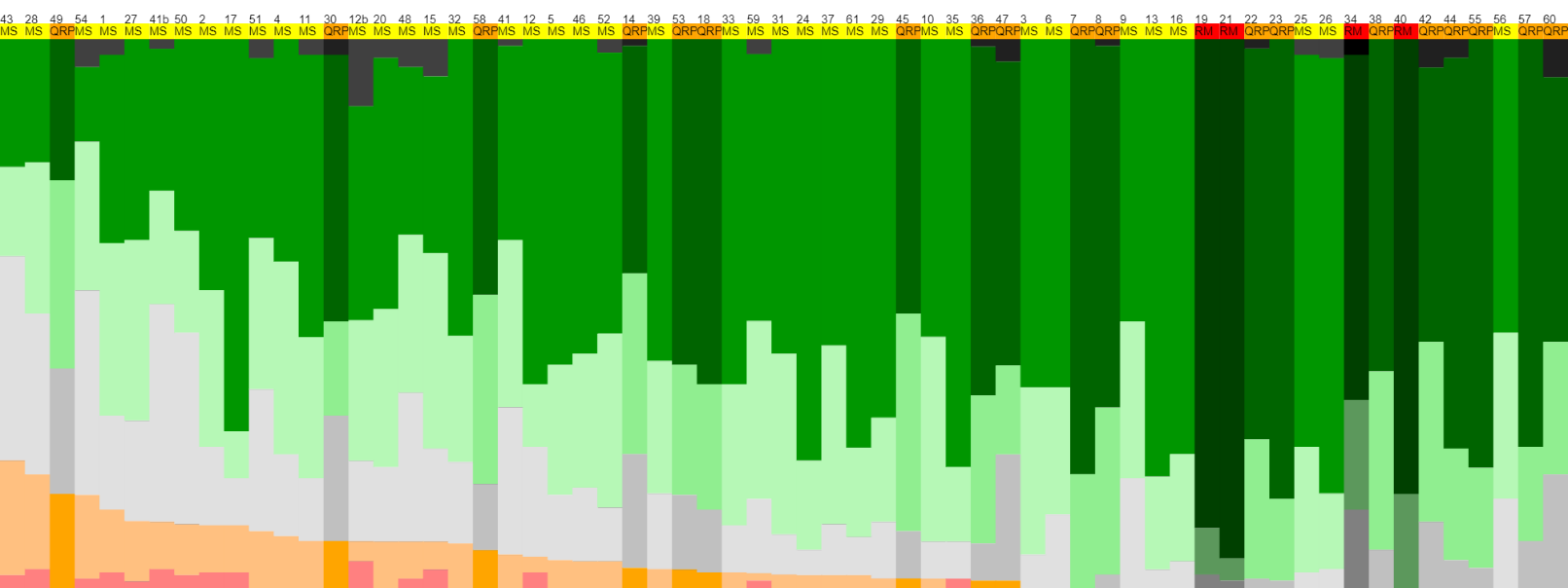
Figure s1 c&d. Likert scores on relevance of 61 Good Research Practices by the faculty of Medicine (c) and Science (d)**

| **Color** | **Group** | **%** |
| --- | --- | --- |
|  | Rather not say | 2 |
|  | 1. Not at all relevant | 2 |
|  | 2 | 5 |
|  | 3 | 14 |
|  | 4 | 23 |
|  | 5. Completely relevant | 53 |

| **Color** | **Group** | **%** |
| --- | --- | --- |
|  | Rather not say | 1 |
|  | 1. Not at all relevant | 1 |
|  | 2 | 4 |
|  | 3 | 13 |
|  | 4 | 24 |
|  | 5. Completely relevant | 56 |

**
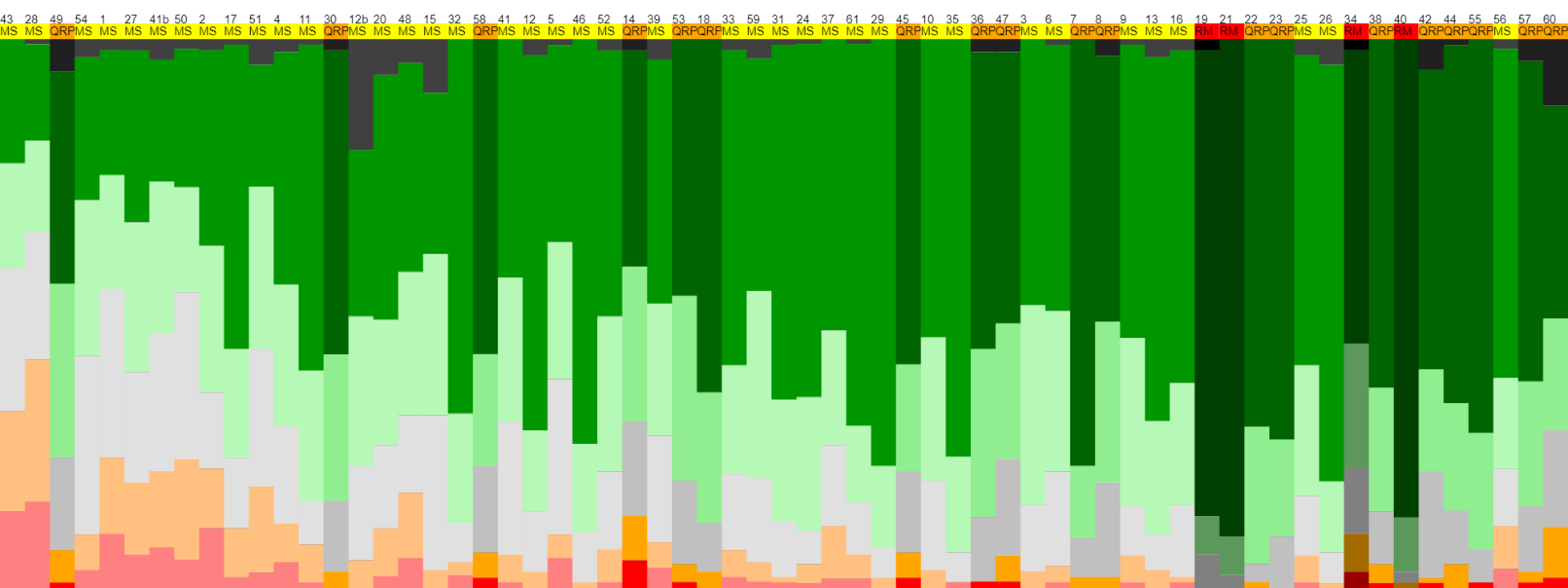
**

**
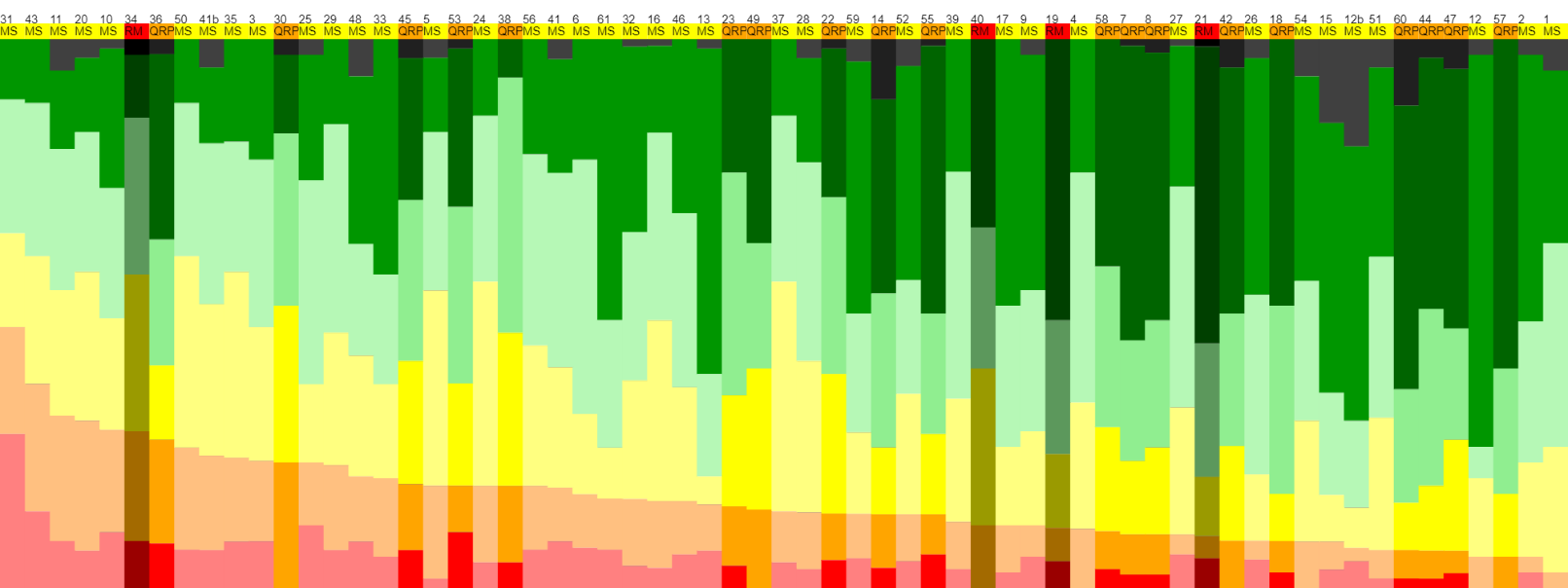
Figure s1 e&f. Likert scores on relevance of 61 Good Research Practices by the faculty of Medicine (e) and Science (f)**

| **Color** | **Group** | **%** |
| --- | --- | --- |
|  | Rather not say | 3 |
|  | 1. Never | 33 |
|  | 2 | 28 |
|  | 3 | 20 |
|  | 4 | 11 |
|  | 5. All the time | 5 |

| **Color** | **Group** | **%** |
| --- | --- | --- |
|  | Rather not say | 7 |
|  | 1. Never | 32 |
|  | 2 | 28 |
|  | 3 | 18 |
|  | 4 | 9 |
|  | 5. All the time | 5 |

**
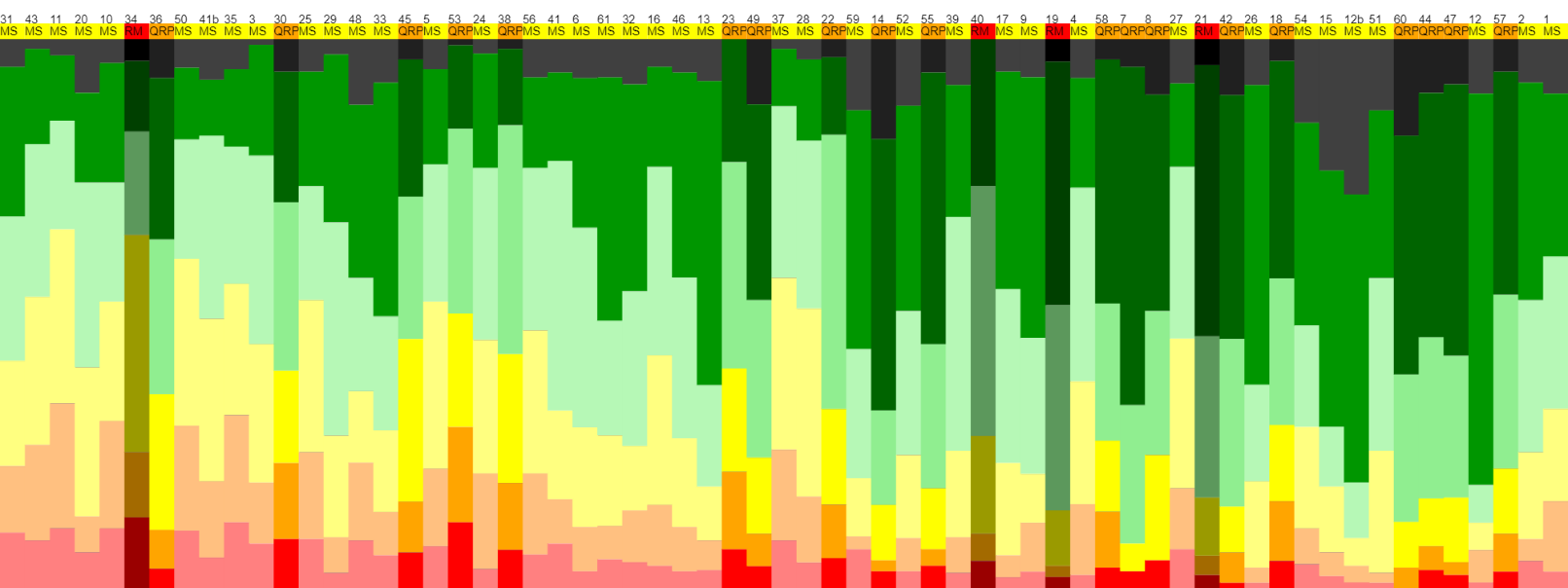
**

**Figure s1 g&h. Likert scores on Seriousness of 61 Good Research Practices by the faculty of Medicine (g) and Science (h)**

| **Color** | **Group** | **%** |
| --- | --- | --- |
|  | Rather not say | 6 |
|  | Minor shortcoming | 25 |
|  | Questionable research practice | 43 |
|  | Research misconduct | 26 |

| **Color** | **Group** | **%** |
| --- | --- | --- |
|  | Rather not say | 4 |
|  | Minor shortcoming | 24 |
|  | Questionable research practice | 45 |
|  | Research misconduct | 28 |

**
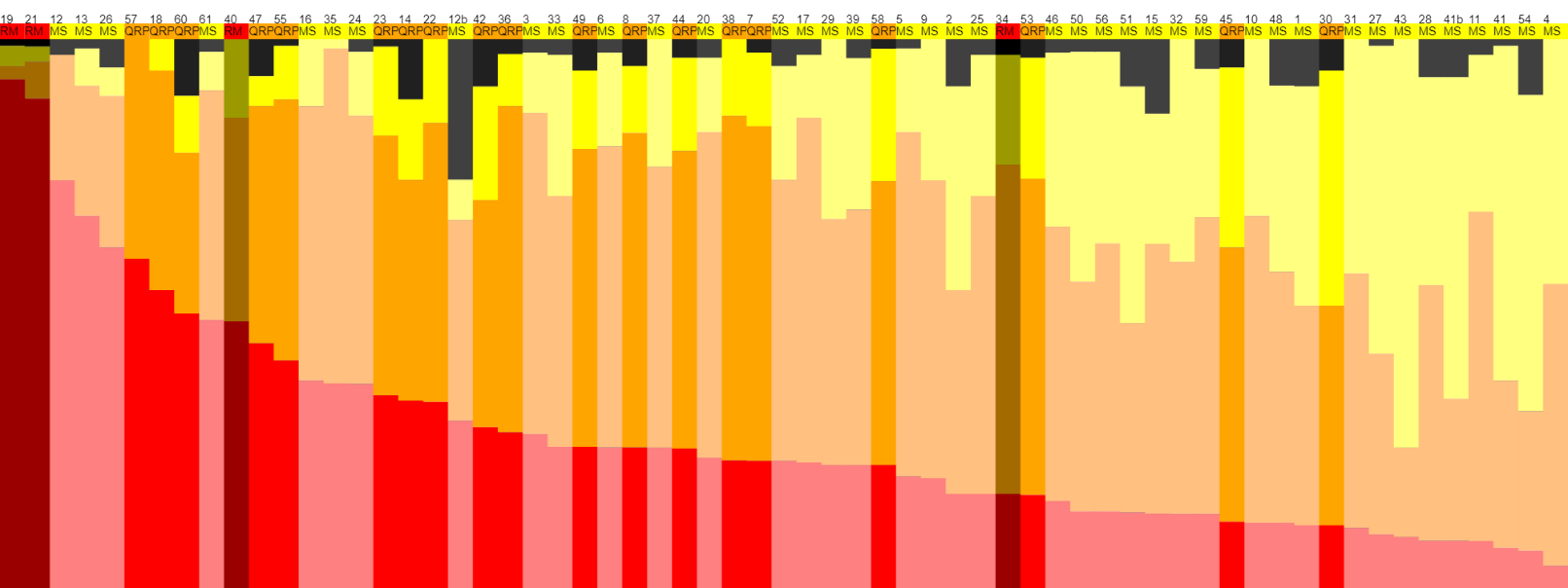

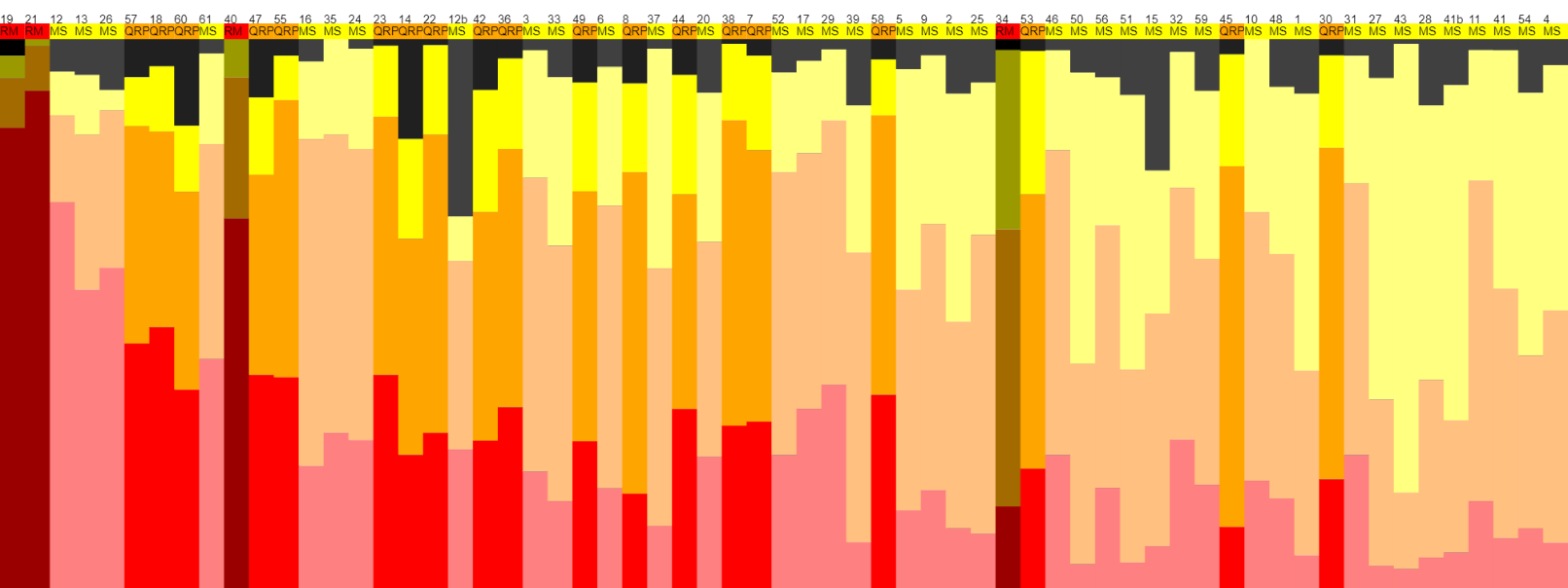
**

*Stacked bar charts display subgroup analyses of the 61 standards for good research practices from the Netherlands code of conduct on research integrity 2018, split by faculty (Medicine and Science). The x-axis lists the practices, and the y-axis shows the percentage of responses per rating. Colored segments within each bar represent ratings arranged by overall score distribution (clarity, relevance, frequency, and seriousness). Labels below each bar indicate if a practice is a minor shortcoming (MS), a questionable research practice (QRP), or research misconduct (RM); bar shading reflects these classifications (light for MS, medium for QRP, dark for RM). Each bar is marked with its corresponding Dutch code number, with standards 12 and 41 divided into “a” and “b”, making 63 evaluation points per chart.*

**Table s1. Subgroup characteristics based on faculty**

| **Variable** | **Groups →** | **Faculty of Medicine** | **Faculty of Science** |  |
| --- | --- | --- | --- | --- |
| Age (years) | \| **Measurement** \| **n = 111*** \| **n = 170*** \| \| --- \| --- \| --- \| \| Median [IQR] \| 28 [26 - 29] \| 26 [25 - 29] \| \| Missing \| 5% (n=6) \| 15% (n=30) \| | | | |
| Survey time (minutes) | \| **Measurement** \| **n = 117*** \| **n = 200*** \| \| --- \| --- \| --- \| \| Median [IQR] \| 32 [21 - 51] \| 35 [23 - 56] \| \| Missing \| 0% (n=0) \| 0% (n=0) \| | | | |
| Mobility | \| **Group** \| **n = 117  % ( n)** \| **n = 200  % ( n)** \| \| --- \| --- \| --- \| \| Fully Educated in the Netherlands \| 69 (81) \| 40 (80) \| \| Education in Europe \| 7 ( 8) \| 26 (51) \| \| Education partially in Europe \| 5 ( 6) \| 12 (23) \| \| Education outside Europe \| 11 (13) \| 21 (41) \| \| Prefer not to disclose \| 6 ( 7) \| ** \| | | | |
| Type of work | \| **Group** \| **% ( n)** \| **% ( n)** \| \| --- \| --- \| --- \| \| Laboratory \| 27 (32) \| 40 (79) \| \| Theoretical \| 5 ( 6) \| 18 (36) \| \| Clinical \| 28 (33) \| ** \| \| Computer modelling \| ** \| 14 (29) \| \| Other \| 5 ( 6) \| ** \| \| Multiple \| 27 (32) \| 22 (44) \| | | | |
| PhD experience | \| **Group** \| **% ( n)** \| **% ( n)** \| \| --- \| --- \| --- \| \| 0-12 months \| 62 (72) \| 40 (80) \| \| 13-24 months \| 21 (24) \| 32 (64) \| \| 25-36 months \| 12 (14) \| 14 (28) \| \| 37-48 months \| ** \| 10 (20) \| | | | |
| Gender | \| **Group** \| **% ( n)** \| **% ( n)** \| \| --- \| --- \| --- \| \| Male \| 31 (36) \| 56 (111) \| \| Female \| 69 (81) \| 42 ( 84) \| | | | |
| Prior training | \| **Group** \| **% ( n)** \| **% ( n)** \| \| --- \| --- \| --- \| \| None \| 44 (51) \| 46 (91) \| \| 2 hours or less \| 17 (20) \| 29 (58) \| \| Over 2 hours \| 36 (42) \| 25 (49) \| | | | |
| * Total non-missing values ** Groups with less than 5% of observations in the subgroup are not shown  IQR: interquartile range | | | | |

*This table compares the demographic characteristics of participants from the faculties of Medicine and Science who evaluated the standards for good research practices in “The Netherlands Code of Conduct for Research Integrity 2018”. Mobility reflects combined information on the country of origin and education (bachelor’s and master’s degrees).The survey time indicates the duration to complete the entire questionnaire.*

**Table s2. Overview of all good research practices and outcome values**

| **Number & Text** | **Phase** | **Outcomes** |
| --- | --- | --- |
| 1: Consider the interests of science and scholarship and/or society when determining the subject and structure of your research. | Design | Clarity: 4.1  Relevance: 3.4  Frequency: 2.0  Seriousness: 1.4 |
| 2: Conduct research that can be of scientific, scholarly and/or societal relevance. | Design | Clarity: 4.3  Relevance: 3.7  Frequency: 1.8  Seriousness: 1.5 |
| 3: Do not make unsubstantiated claims about potential results. | Design | Clarity: 4.5  Relevance: 4.4  Frequency: 2.5  Seriousness: 2.0 |
| 4: Take into account the latest scientific and scholarly insights. | Design | Clarity: 4.4  Relevance: 4.0  Frequency: 2.2  Seriousness: 1.6 |
| 5: Make sure that your research design can answer the research question. | Design | Clarity: 4.2  Relevance: 4.0  Frequency: 2.5  Seriousness: 1.8 |
| 6: Ensure that the methods you employ are well justified. | Design | Clarity: 4.4  Relevance: 4.3  Frequency: 2.1  Seriousness: 1.9 |
| 7: If the research is conducted on commission and/or funded by third parties, always specify who the commissioning party and/or funding body is. | Design | Clarity: 4.8  Relevance: 4.7  Frequency: 1.6  Seriousness: 2.1 |
| 8: Be open about the role of external stakeholders and possible conflicts of interest. | Design | Clarity: 4.3  Relevance: 4.4  Frequency: 1.8  Seriousness: 1.9 |
| 9: In research with external partners, make clear written agreements about research integrity and related matters such as intellectual property rights. | Design | Clarity: 4.5  Relevance: 4.3  Frequency: 1.8  Seriousness: 1.9 |
| 10: As necessary, describe how the collected research data are organized and classified so that they can be verified and reused. | Design | Clarity: 4.4  Relevance: 4.4  Frequency: 2.6  Seriousness: 1.8 |
| 11: As far as possible, make research findings and research data public subsequent to completion of the research. If this is not possible, establish valid reasons for their non-disclosure. | Design | Clarity: 4.6  Relevance: 4.3  Frequency: 2.9  Seriousness: 1.9 |
| 12a: In the event of an investigation into alleged research misconduct, make all relevant research and data available for verification subject to the confidentiality safeguards established by the board of the institution. | Design | Clarity: 4.4  Relevance: 4.4  Frequency: 1.3  Seriousness: 2.6 |
| 12b: In highly exceptional cases, there may be compelling reasons for components of the research, including data, not to be disclosed to an investigation into alleged research misconduct. Such cases must be recorded and the consent of the board of the institution must be obtained prior to using the components and/or data in question in the scientific or scholarly research. They must also be mentioned in any results published. | Design | Clarity: 3.4  Relevance: 3.3  Frequency: 1.2  Seriousness: 1.6 |
| 13: Ensure that the required permissions are obtained and that, where necessary, an ethical review is conducted. | Design | Clarity: 4.6  Relevance: 4.5  Frequency: 1.7  Seriousness: 2.4 |
| 14: Accept only research assignments that can be undertaken in accordance with the standards in this Code. | Design | Clarity: 4.0  Relevance: 3.9  Frequency: 1.6  Seriousness: 1.8 |
| 15: Enter into joint research with a partner not affiliated with an institution which has adopted this or a comparable Code only if there is sufficient confidence that your own part of the research can be conducted in compliance with this Code and the joint research results meet generally accepted principles of integrity in research. | Design | Clarity: 3.6  Relevance: 3.6  Frequency: 1.4  Seriousness: 1.5 |
| 16: Conduct your research accurately and with precision. | Conduct | Clarity: 4.4  Relevance: 4.5  Frequency: 2.4  Seriousness: 2.1 |
| 17: Employ research methods that are scientific and/or scholarly. | Conduct | Clarity: 4.0  Relevance: 4.2  Frequency: 1.8  Seriousness: 2.1 |
| 18: Make sure that the choice of research methods, data analysis, assessment of results and consideration of possible explanations is not determined by non-scientific or non-scholarly (e.G. Commercial or political) interests, arguments or preferences. | Conduct | Clarity: 4.6  Relevance: 4.5  Frequency: 2.0  Seriousness: 2.3 |
| 19: Do not fabricate data or research results and do not report fabricated material as if it were fact. | Conduct | Clarity: 4.7  Relevance: 4.8  Frequency: 1.8  Seriousness: 2.8 |
| 20: Do justice to all research results obtained. | Conduct | Clarity: 3.9  Relevance: 3.9  Frequency: 2.4  Seriousness: 1.9 |
| 21: Do not remove or change results without explicit and proper justification. Do not add fabricated data during the data analysis. | Conduct | Clarity: 4.9  Relevance: 4.9  Frequency: 1.7  Seriousness: 2.9 |
| 22: Ensure that sources are verifiable. | Conduct | Clarity: 4.6  Relevance: 4.6  Frequency: 2.3  Seriousness: 2.1 |
| 23: Describe the data collected for and/or used in your research honestly, scrupulously and as transparently as possible. | Conduct | Clarity: 4.7  Relevance: 4.7  Frequency: 2.4  Seriousness: 2.2 |
| 24: Manage the collected data carefully and store both the raw and processed versions for a period appropriate for the discipline and methodology at issue. | Conduct | Clarity: 4.5  Relevance: 4.5  Frequency: 2.5  Seriousness: 2.1 |
| 25: Contribute, where appropriate, towards making data findable, accessible, interoperable and reusable in accordance with the FAIR principles. | Conduct | Clarity: 4.2  Relevance: 4.3  Frequency: 2.5  Seriousness: 1.7 |
| 26: Take into consideration the interests of any humans and animals involved, including test subjects, as well as any risks to the researchers and the environment, while always observing the relevant statutory regulations and codes of conduct. | Conduct | Clarity: 4.5  Relevance: 4.6  Frequency: 1.6  Seriousness: 2.4 |
| 27: Keep your own level of expertise up to date. | Conduct | Clarity: 4.4  Relevance: 3.7  Frequency: 2.3  Seriousness: 1.4 |
| 28: Take on only those tasks that fall within your area of expertise. | Conduct | Clarity: 4.2  Relevance: 3.1  Frequency: 2.4  Seriousness: 1.4 |
| 29: Do justice to everyone who contributed to the research and to obtaining and/or processing the data. | Reporting results | Clarity: 4.7  Relevance: 4.6  Frequency: 2.2  Seriousness: 2.1 |
| 30: Ensure a fair allocation and ordering of authorship, in line with the standards applicable within the discipline(s) concerned. | Reporting results | Clarity: 4.4  Relevance: 4.2  Frequency: 2.4  Seriousness: 1.9 |
| 31: All authors must have made a genuine intellectual contribution to at least one of the following elements: the design of the research, the acquisition of data, its analysis or the interpretation of findings. | Reporting results | Clarity: 4.7  Relevance: 4.5  Frequency: 2.8  Seriousness: 1.8 |
| 32: All authors must have approved the final version of the research product. | Reporting results | Clarity: 4.9  Relevance: 4.4  Frequency: 2.0  Seriousness: 1.8 |
| 33: All authors are fully responsible for the content of the research product, unless otherwise stated. | Reporting results | Clarity: 4.7  Relevance: 4.2  Frequency: 2.0  Seriousness: 1.8 |
| 34: Present sources, data and arguments in a scrupulous way. | Reporting results | Clarity: 4.2  Relevance: 4.2  Frequency: 2.8  Seriousness: 1.8 |
| 35: Be transparent about the method and working procedure followed and record them where relevant in research protocols, logs, lab journals or reports. The line of reasoning must be clear and the steps in the research process must be verifiable. This usually means that the research must be described in sufficient detail for it to be possible to replicate the data collection and its analysis. | Reporting results | Clarity: 4.7  Relevance: 4.7  Frequency: 2.7  Seriousness: 2.2 |
| 36: Be explicit about any relevant unreported data that has been collected in accordance with the research design and could support conclusions different from those reported. | Reporting results | Clarity: 4.4  Relevance: 4.4  Frequency: 2.2  Seriousness: 2.1 |
| 37: Be clear about results and conclusions, as well as their scope. | Reporting results | Clarity: 4.5  Relevance: 4.2  Frequency: 2.7  Seriousness: 1.8 |
| 38: Be explicit about uncertainties and contraindications, and do not draw unsubstantiated conclusions. | Reporting results | Clarity: 4.6  Relevance: 4.5  Frequency: 2.5  Seriousness: 2.1 |
| 39: Be explicit about serious alternative insights that could be relevant to the interpretation of the data and the research results. | Reporting results | Clarity: 4.4  Relevance: 4.1  Frequency: 2.1  Seriousness: 1.7 |
| 40: When making use of other people’s ideas, procedures, results and text, do justice to the research involved and cite the source accurately. | Reporting results | Clarity: 4.9  Relevance: 4.8  Frequency: 2.1  Seriousness: 2.5 |
| 41a: Avoid unnecessary reuse of previously published texts of which you were the author or co-author. Be transparent about reuse by citing the original publication. | Reporting results | Clarity: 4.5  Relevance: 4.0  Frequency: 2.3  Seriousness: 1.5 |
| 41b: Avoid unnecessary reuse of previously published texts of which you were the author or co-author. Such self-citation is not necessary for reuse on a small scale or of introductory passages and descriptions of the method applied. | Reporting results | Clarity: 4.0  Relevance: 3.4  Frequency: 2.5  Seriousness: 1.3 |
| 42: Always provide references when reusing research material that can be used for meta-analysis or the analysis of pooled data. | Reporting results | Clarity: 4.2  Relevance: 4.1  Frequency: 1.6  Seriousness: 1.9 |
| 43: Avoid unnecessary references and do not make the bibliography unnecessarily long. | Reporting results | Clarity: 4.4  Relevance: 3.3  Frequency: 2.7  Seriousness: 1.2 |
| 44: Be open and complete about the role of external stakeholders, commissioning parties, funding bodies, possible conflicts of interest and relevant ancillary activities. | Reporting results | Clarity: 4.6  Relevance: 4.5  Frequency: 1.7  Seriousness: 2.0 |
| 45: As far as possible, make research findings and research data public subsequent to completion of the research. If this is not possible, establish the valid reasons for this. | Reporting results | Clarity: 4.6  Relevance: 4.3  Frequency: 2.3  Seriousness: 1.8 |
| 46: Be honest and scrupulous as an assessor or peer reviewer, and explain your assessment. | Assessment and peer review | Clarity: 4.6  Relevance: 4.5  Frequency: 2.1  Seriousness: 1.9 |
| 47: Do not use information acquired in the context of an assessment without explicit consent. | Assessment and peer review | Clarity: 4.1  Relevance: 4.1  Frequency: 1.7  Seriousness: 2.2 |
| 48: Do not use the system of peer review to generate additional citations for no apparent reason, with the aim of increasing your own or other people’s citation scores (‘citation pushing’). | Assessment and peer review | Clarity: 4.0  Relevance: 3.7  Frequency: 2.2  Seriousness: 1.7 |
| 49: Refrain from making an assessment if any doubts could arise regarding your independence (for example, because of possible commercial or financial interests). | Assessment and peer review | Clarity: 4.0  Relevance: 3.8  Frequency: 1.9  Seriousness: 1.9 |
| 50: Refrain from making an assessment outside your area of expertise, or do so only in general terms. | Assessment and peer review | Clarity: 4.1  Relevance: 3.5  Frequency: 2.7  Seriousness: 1.5 |
| 51: Be generous in cooperating with internal and external reviews of your own research. | Assessment and peer review | Clarity: 3.9  Relevance: 3.5  Frequency: 1.8  Seriousness: 1.4 |
| 52: Do not establish a journal that does not apply the required standards of quality to its publications, and do not cooperate with any such journal. | Assessment and peer review | Clarity: 4.3  Relevance: 4.2  Frequency: 1.9  Seriousness: 1.9 |
| 53: Be honest in public communication and clear about the limitations of the research and your own expertise. Only communicate to the general public about the research results if there is sufficient certainty about them. | Communication | Clarity: 4.5  Relevance: 4.3  Frequency: 2.6  Seriousness: 1.9 |
| 54: Be open and honest about your role in the public debate and about the nature and status of your participation in it. | Communication | Clarity: 3.7  Relevance: 3.4  Frequency: 1.8  Seriousness: 1.4 |
| 55: Be open and honest about potential conflicts of interest. | Communication | Clarity: 4.7  Relevance: 4.7  Frequency: 1.8  Seriousness: 2.3 |
| 56: As a supervisor, principal investigator, research director or manager, provide for an open and inclusive culture in all phases of research. | All phases | Clarity: 4.4  Relevance: 4.3  Frequency: 2.4  Seriousness: 1.8 |
| 57: As a supervisor, principal investigator, research director or manager, refrain from any action which might encourage a researcher to disregard any of the standards in this chapter. | All phases | Clarity: 4.3  Relevance: 4.3  Frequency: 1.8  Seriousness: 2.3 |
| 58: Do not delay or hinder the work of other researchers in an inappropriate manner. | All phases | Clarity: 4.5  Relevance: 4.2  Frequency: 2.0  Seriousness: 2.0 |
| 59: Call attention to other researchers’ non-compliance with the standards as well as inadequate institutional responses to non-compliance, if there is sufficient reason for doing so. | All phases | Clarity: 4.1  Relevance: 4.1  Frequency: 1.8  Seriousness: 1.7 |
| 60: In addressing research misconduct, make no accusation that you know or should have known to be incorrect. | All phases | Clarity: 3.6  Relevance: 3.6  Frequency: 1.4  Seriousness: 2.0 |
| 61: Do not make improper use of research funds. | All phases | Clarity: 4.5  Relevance: 4.5  Frequency: 1.8  Seriousness: 2.3 |

*This table lists all the good research practices outlined in Chapter 3 of The Netherlands Code of Conduct for Research Integrity 2018. The "Phase" column indicates the stage of the research process to which each practice applies. The "Mean Score" column presents the average Likert scale ratings. A score of 1 corresponds to "completely unclear," "completely irrelevant," or "never experienced this in my direct environment," while a score of 5 represents "completely clear," "completely relevant," or "experienced this whenever possible." For "Seriousness," a score of 1 denotes that non-adherence is considered a minor shortcoming, 2 indicates a questionable research practice, and 3 signifies research misconduct.*
